# Guillain-Barré Syndrome Following the First Dose of Inactivated SARS-CoV-2 Vaccine, BBIBP-CorV

**DOI:** 10.7759/cureus.33952

**Published:** 2023-01-18

**Authors:** Lanka Wijekoon, Chamara Sarathchandra, Sisira Siribaddana

**Affiliations:** 1 Department of Medicine, Rajarata University of Sri Lanka, Anuradhapura, LKA

**Keywords:** nerve conduction studies (ncs), bifacial weakness with paraesthesia, sinopharm bbibp, covid-19 vaccine complication, guillain barre syndrome (gbs)

## Abstract

We present a case report of Guillain-Barré syndrome (GBS) following inactivated whole virus severe acute respiratory syndrome coronavirus 2 (SARS-CoV-2) vaccine, BBIBP-CorV. A man presented with paresthesia in both upper and lower limbs with bifacial weakness, onset 18 days after receiving the first BBIBP-CorV vaccine. A bifacial palsy with a paresthesia variant of GBS was diagnosed, and the patient was treated with intravenous immunoglobulin, arresting the progression of neurological symptoms. Clinicians need to be aware of the possibility of GBS following vaccination with BBIBP-CorV, an inactivated SARS-CoV-2 vaccine.

## Introduction

In Sri Lanka, the coronavirus disease 2019 (COVID-19) vaccination began in January 2021. Currently, the country uses five COVID-19 vaccines. As of 30 October 2022, Sri Lanka had administered 14.75 million vaccines, with 76% of them being BBIBP-CorV. Guillain-Barré syndrome (GBS) has occurred after vaccination with ChAdOx1-S, Ad26.COV2.S, BNT162b2, mRNA-1273, and BBIBP-CorV [1-5]. We describe a patient with GBS following the first dose of the BBIBP-CorV vaccine.

## Case presentation

A 41-year-old male with type 2 diabetes presented with numb legs and pain in his upper back and was admitted to a teaching hospital. He was on metformin 500mg daily for the past year. Pain and numbness started 18 days after receiving the first dose of BBIBP-CorV. Numbness worsened over the next week to involve all four limbs, and by the ninth day, he developed bilateral facial weakness. He did not report recent respiratory or diarrheal disease.

He had a glove and stocking-type sensory loss to touch, up to the elbow and knee level, with intact pain, temperature, and joint position sensation. All four-limb muscle power was normal, with a 5/5 Medical Research Council scale with diminished deep tendon reflexes. Cerebellar, autonomic, and respiratory functions were preserved, and except for isolated bilateral lower motor neuron-type facial palsy other cranial nerves, including bulbar functions, were intact. The patient had a disability scale of 2 on the GBS disability scale [6].

Cerebral-spinal fluid (CSF) analysis on day 13 of the illness revealed a cell protein dissociation, with elevated CSF protein (62.2 g/dL) and a normal cell count of five lymphocytes per mm^3^ corroborating the GBS diagnosis. On the same day, a nerve conduction study (NCS) was performed (Tables 1, 2) and revealed reduced motor nerve conduction velocities in bilateral peroneal nerves and prolonged distal motor latencies in the bilateral peroneal and right tibial nerve. The sensory response in the right ulnar nerve was not detected, but the bilateral sural sensory response was preserved. We could not arrange a follow-up study.

**Table 1:** Motor nerve conduction study *Upper limits of the normal values are mentioned within brackets, #Lower limits of the normal values are mentioned within brackets. ADM: Abductor digiti minimi; EDB: extensor digitorum brevis; AH: adductor hallucis

Nerve/Sites	Latency (ms)*	Amplitude# (mV)	Duration (ms)	Relative amplitude%	Segments	Distance (mm)	Latency Difference (ms)		Velocity# (m/s)
Right Ulnar – ADM									
Wrist	4.17(3.1)	4.1(7.4)	8.49	100	Wrist-ADM	80			
Below Elbow	9.27(3.1)	0.8(7.4)	9.06	19.5	Wrist-Below Elbow	270	5.10		53(54)
Left Ulnar – ADM									
Wrist	4.32(3.1)	3.3(7.4)	7.19	100	Wrist-ADM	80			
Below Elbow	10.16(3.1)	3.2(7.4)	8.59	96.1	Wrist-Below Elbow	270	5.83		46(54)
Right Peroneal - EDB									
Ankle	8.96(4.4)	0.3(3.2)	9.48	100	Ankle-EDB	80			
Fibular Head	25.4(4.4)	0.7(3.2)	7.60	243	Fibular Head- Ankle	350	16.51		21(45)
Left Peroneal - EDB									
Ankle	6.72(4.4)	0.8(3.2)	16.20	100	Ankle-EDB	80			
Fibular Head	19.74(4.4)	0.5(3.2)	14.06	63.9	Fibular Head- Ankle	340	13.02		26(45)
Right Tibial – AH									
Ankle	10.21(5.0)	0.4(5.7)	19.95	100	Ankle-AH	80			

**Table 2:** Sensory nerve conduction study *Upper limits of the normal values are mentioned within brackets, #Lower limits of the normal values are mentioned within brackets.

Nerve/Sites	Recording site	Onset Latency* (ms)	Peak Latency*(ms)	Amplitude# (mV)	Segments	Distance (mm)	Velocity# (m/s)
Right Ulnar – Fifth digit (Antidromic)							
Wrist	Fifth digit	Not recorded	Not recorded	Not recorded	Wrist Fifth digit	140	Not recorded
Right Sural – Ankle (Calf)							
Calf	Ankle	1.98(3.0)	2.71(3.8)	7.7(12.0)	Calf-Ankle	120	61(44)
Left Sural – Ankle (Calf)							
Calf	Ankle	1.41(3.0)	2.08(3.8)	7.1(12.0)	Calf-Ankle	120	85(44)

Serum sodium was low at 123 mmol/l, and serum osmolarity was normal at 275 mOsm/kg. The urine osmolarity was abnormally high at 400 mOsm/kg. The urine sodium level was 45 mmol/l, confirming the syndrome of inappropriate antidiuretic hormone production (SIADH), a recognized consequence of GBS. A nasopharyngeal swab was negative for severe acute respiratory syndrome coronavirus 2 (SARS-CoV-2) real-time reverse transcription-polymerase chain reaction. Serum IgM antibodies for SARS-CoV-2 were detected, indicating an antibody response to the BBIBP-CorV vaccine. 

Initially, the patient had numbness in both legs, but upper limbs and cranial nerves were spared. At that time, the differentials were GBS and other causes of acute predominantly sensory peripheral neuropathy, such as paraproteinemic and paraneoplastic neuropathy. However, with the rapid symmetrical involvement of upper limbs and bilateral lower motor neuron facial nerve palsy, a non-length-dependent polyradiculopathy such as GBS was considered more likely. With the given clinical picture of bifacial weakness, paresthesia and hyporeflexia following the vaccination and with supportive CSF and nerve conduction tests, the bifacial weakness with paresthesia (BFP) variant of GBS was diagnosed according to the National Institute of Neurological Disorders and Stroke criteria for GBS [7]. 

He was admitted to the high-dependency unit for observation, and clinical parameters were monitored with prophylaxis for deep venous thrombosis. Intravenous immunoglobulin (IVIG) was given at a dose of 0.4g/Kg for five days. We conservatively treated hyponatremia due to SIADH with the restriction of fluid intake to 1000ml per day for three days, with improvement.

After IVIG, the progression of neurological symptoms stopped, and bilateral facial weakness was present on discharge. The patient was advised not to take the second dose of the BBIBP-CorV. The patient gradually improved over three weeks, and neurological symptoms resolved fully. He is currently active and in good health.

## Discussion

COVID-19 vaccination prevents death. In Sri Lanka, five different types of vaccinations are currently in use. BBIBP-CorV, developed by the Beijing Institution of Biological Products, is the most frequent vaccine used in Sri Lanka. It is an inactivated whole virus vaccine with an efficacy of 79% against symptomatic COVID-19 infection and hospitalization [8]. 

COVID-19 vaccinations have been linked to severe adverse events. Vaccine-induced immune thrombocytopenic thrombosis was identified following the adenovirus viral vector vaccines, particularly with ChAdOx1-S [9]. Myocarditis was recognized after the mRNA vaccines [10]. However, severe adverse events were not reported after BBIBP-CorV [11].

GBS is a peripheral nerve disease due to immunological sequela from previous respiratory, gastrointestinal, bacterial, or viral infection. GBS has been linked to immunizations [12]. Pathophysiologically, antibodies produced by a vaccine could cross-react with the protein found in the myelin sheath of peripheral neurons [13].

In a recent systematic review of post COVID-19 vaccination with GBS, 88 patients were identified, and ChAdOx1-S was the associated vaccine in 59%, followed by BNT162b2 (22.7%) [14]. In 79.5% of the cases, GBS occurred following the first dose of vaccination [14]. GBS developed in six patients after the inactivated BBIBP-CorV [5,15,16]. Four presented following the first dose, one after the second, and the other after the third booster dose. Three had classic sensorimotor GBS, and the others had pure motor variant GBS. Three patients had GBS with the electrophysiological subtype of acute motor axonal neuropathy, two had the acute motor and sensory axonal neuropathy (AMSAN), and the other had the acute inflammatory demyelinating polyneuropathy (AIDP) subtype. The BFP variant of GBS was seen in 15.9% of the patients after COVID-19 vaccination [14], while it is generally seen in less than 5% of other GBS patients [7]. The most common electrophysiological subtype of GBS following COVID-19 vaccination was AIDP, which was seen in 43.2%, and AMSAN was seen in 10.2% [14]. 

Our patient experienced bilateral symmetric lower extremity numbness 18 days after receiving the first dose of inactivated SARS-CoV-2 vaccine, BBIBP-CorV and subsequently developed bifacial weakness with no limb weakness. In a 2009 retrospective case analysis of 22 patients with the BFP variant of GBS, only four patients had limb weakness, and 14 had demyelination [17]. A 2022 scoping review of post-COVID-19 vaccination found 18 patients with a BFP variant [18]. Out of 13 available NCSs, five had demyelination. Our patient had electrophysiological data suggestive of focal segmental demyelinating sensory and motor polyneuropathy.

Prominent bifacial weakness with paresthesia in the lower limbs in our patient could point to a distinct clinical form of GBS after COVID-19 vaccinations. However, no BFP variant of GBS was reported after the BBIBP-CorV vaccine. He had SIADH associated with GBS, a known association of GBS and is an indicator of poor prognosis [19].

Aside from the temporal relationship and the lack of prior respiratory or diarrheal illness, there is no substantial proof that the BBIBP-CorV caused GBS. More research with effective postvaccination surveillance systems is required to establish causation. The lack of other case reports of GBS or other neurological after-effects of the BBIBP-CorV vaccine in Sri Lanka may be due to the inadequate surveillance system to detect adverse effects after immunization. It is particularly true for vaccines such as BBIBP-CorV, administered mainly in countries with poor record keeping. 

## Conclusions

Inactivated SARS-CoV-2 BBIBP-CorV can cause the BFP variant of GBS like other vaccines. This variant of GBS may be more common after COVID-19 vaccination. Surveillance systems to detect adverse effects after immunization need improvement in developing countries.

## Figures and Tables

**Figure 1:**
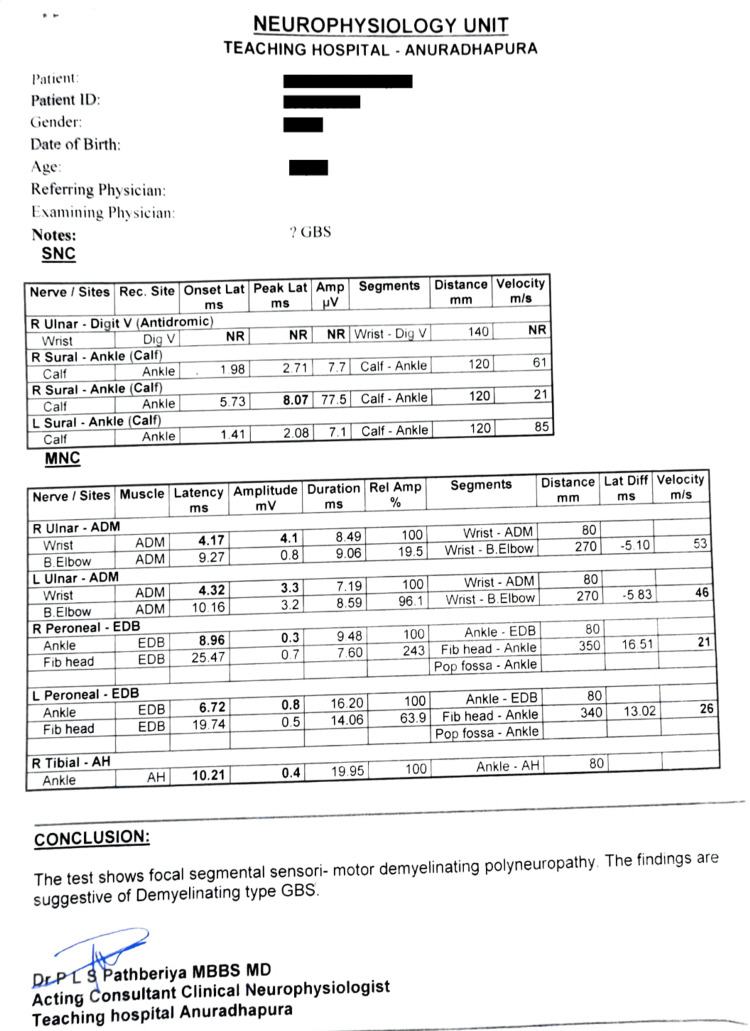
Original nerve conduction study report.
